# Uncoupling Time and Space in the Collinear Regulation of *Hox* Genes

**DOI:** 10.1371/journal.pgen.1000398

**Published:** 2009-03-06

**Authors:** Patrick Tschopp, Basile Tarchini, François Spitz, Jozsef Zakany, Denis Duboule

**Affiliations:** 1 National Research Centre “Frontiers in Genetics”, Department of Zoology and Animal Biology, University of Geneva, Sciences III, Geneva, Switzerland; 2 School of Life Sciences, Federal Institute of Technology (EPFL), Lausanne, Switzerland; Harvard Medical School, United States of America

## Abstract

During development of the vertebrate body axis, *Hox* genes are transcribed sequentially, in both time and space, following their relative positions within their genomic clusters. Analyses of animal genomes support the idea that *Hox* gene clustering is essential for coordinating the various times of gene activations. However, the eventual collinear ordering of the gene specific transcript domains in space does not always require genomic clustering. We analyzed these complex regulatory relationships by using mutant alleles at the mouse *HoxD* locus, including one that splits the cluster into two pieces. We show that both positive and negative regulatory influences, located on either side of the cluster, control an early phase of collinear expression in the trunk. Interestingly, this early phase does not systematically impact upon the subsequent expression patterns along the main body axis, indicating that the mechanism underlying temporal collinearity is distinct from those acting during the second phase. We discuss the potential functions and evolutionary origins of these mechanisms, as well as their relationship with similar processes at work during limb development.

## Author Summary

*Hox* genes encode proteins that control embryonic development along the head-to-tail axis. These genes are clustered in one site on the chromosome and their respective positions within the cluster determine their time and place of activation. Here, by using a large set of targeted mutations disturbing the integrity of the gene cluster, we show that the spatial organization of expression domains does not directly depend upon the timing of activation as was previously suggested. This uncoupling between space and time in the regulation of these *Hox* genes coincides with the existence of two major phases of regulation. The first is time-dependent and involves global regulatory influences, located outside the gene cluster, whereas the second relies upon more local regulatory elements, likely interspersed between the genes, inside the cluster. These results provide the bases for future analyses of collinear mechanisms and indicate that different types of collinearities are not necessarily related, neither in function, nor in their evolutionary histories.

## Introduction

*Hox* genes play essential roles in patterning during the development of metazoans. In many species, they are found clustered in the genome, such as in vertebrates, which contain four *Hox* gene clusters (*HoxA* to *HoxD*), due to the additional two rounds of genome amplification that accompanied their emergence from early chordates. These genes are required to confer regional identities along the rostral to caudal body axis, a task that mostly depends upon particular combinations of HOX proteins found at a given anterior-posterior level, since genes of all four clusters are expressed in largely overlapping domains [1],[2]. In mouse, combined mutations produce drastic effects on the specification of extended body regions, as exemplified by the inactivation of genes belonging to the paralogy group 10, which triggered the appearance of ectopic ribs along the lumbar and sacral regions [3]. Therefore, a precise spatial distribution of these transcription factors must be orchestrated so as to ensure proper specification.

These regionalized expression domains are in part controlled at a transcriptional level, by using an intrinsic property of the gene clusters, conserved from insects to vertebrates and referred to as spatial collinearity [4]–[7]: the order of genes along the chromosome correlates with their successive anterior limits of expression along the body axis. Vertebrates display yet another type of collinearity whereby the relative timing of *Hox* gene activation during development follows the gene sequence, such that genes lying at one extremity of a cluster are activated earlier and more rostrally than genes located near the other extremity [8],[9]. Murine *Hoxd* genes thus become activated in the most posterior part of the embryo between late embryonic day 7.75 (E7.75) for *Hoxd1* and early E9 for *Hoxd13*. This temporal progression was proposed to be a molecular clock (the ‘*Hox* clock’) controlling the proper timing of axial specification by coordinating the rostral-caudal positions of the various expression boundaries [10]. While this view has found some support in studies of early limb patterning, where a strong correlation exists between the onset of *Hox* gene expression in the incipient limb bud and the extent of expression along the anterior to posterior axis [11], the situation in the developing major body axis appeared more complex.

First, it was noticed early on [12],[13] that *Hox* transgenes could be expressed with rather faithful anterior boundaries, yet not necessarily with the exact expression timing. Secondly, targeted *Hox* cluster modifications *in vivo*, which changed the timing of activation, induced patterning problems even without modification of the late expression boundaries [14]. Finally, spatial collinearity is still observed, to some extent, in animals where *Hox* genes are not clustered such as the larvacean Oikopleura [15]. Altogether, these observations suggest that, while gene clustering may be an absolute requirement for implementing the temporal sequence of activation (see [10],[16],[17]), important aspects of spatial regulation do not require tight clustering.

So far, the relationships between the time of *Hox* gene activation and their expression territories have been best documented in developing limbs (e.g. [11],[18]), i.e. in structures which do not obligatorily implement the same regulatory mechanisms than those at work in the developing trunk, to activate this gene family (see [19],[20]). In this work, we assess the importance of genomic clustering for the temporal and spatial collinear regulations of *Hox* genes during the development of the major body axis. We use mutant mice where the *HoxD* cluster is split into two independent sub-clusters, as well as a collection of deletion and duplication alleles. We show that temporal activation relies upon a balance between a repressive activity, mediated *via* the centromeric neighborhood of the cluster, and an activating effect mediated by the telomeric region. Remarkably, however, modifications in this early time sequence are not systematically translated into concurrent alterations in the subsequent spatial distribution of transcripts, which mostly depends upon local, interspersed regulatory elements. Consequently, temporal and spatial collinear controls appear to be mechanistically uncoupled.

## Results

### Interruption of Temporal Collinearity

**Figure 1:**
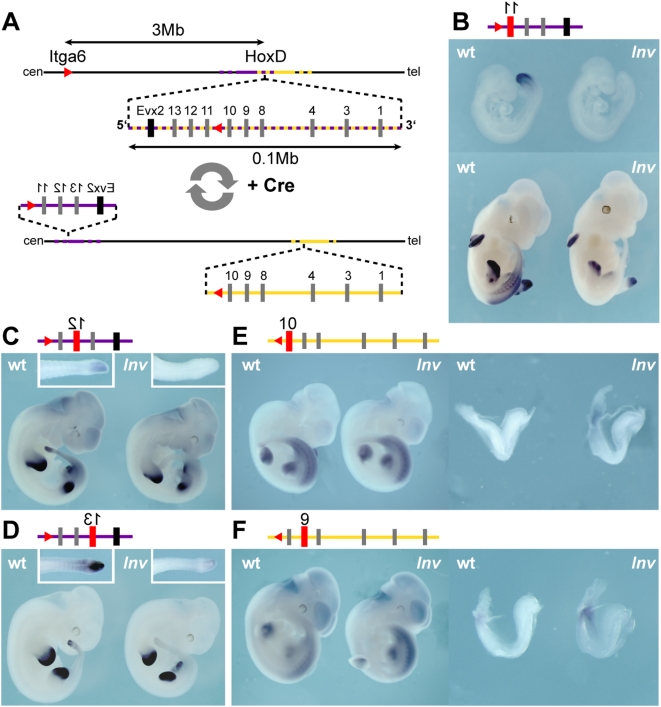
A split *HoxD* cluster reveals both positive and negative regulations for early expression in the developing trunk. (A) The inversion divides the *HoxD* cluster into two sub-clusters. In the wild type configuration, the cluster is under the mixed regulatory influences coming from either the centromeric (purple), or the telomeric (yellow) sides. After targeted inversion (bottom line), 3 Mb separate the centromeric part of the cluster from the remaining ‘anterior’ part. *Hoxd11*, *Hoxd12* and *Hoxd13* are now under the influence of the centromeric neighborhood only (purple), whereas *Hoxd1* to *Hoxd10* are associated with the telomeric region (yellow). Red triangles depict *loxP* sites. (B–F) Each panel is accompanied by a scheme of the respective sub-cluster, and the gene analyzed is shown in red. (B–D) Wild type (left) and mutant (*Inv*; right) embryos hybridized with *Hoxd11*, *Hoxd12* and *Hoxd13* probes. In mutant embryos, *Hoxd11* expression is completely lost from the trunk (B, upper panel), whereas the developing limb and genitalia show the expected wild type pattern at E12.5 (B, lower panel). Similar effects are scored for both *Hoxd12* (C) and *Hoxd13* (D) at E11.5, showing expression in both limb and genital buds, but no detectable signal in the primary body axis. (E, F) Wild type (left) and mutant (right) embryos hybridized for *Hoxd10* and *Hoxd9*, which are located in the other sub-cluster. At E8, both *Hoxd10* and *Hoxd9* show elevated expression in the developing trunk.

We evaluated whether the integrity of a *Hox* gene cluster is essential for temporal collinearity during early trunk development, by using a targeted inversion that splits the *HoxD* cluster into two smaller, independent gene clusters [21]. One of the inversion breakpoints was located between *Hoxd10* and *Hoxd11*, and the other at the *Itga6* (integrin alpha 6) locus, about 3 megabases (Mb) centromeric to *HoxD*. The inversion separates the most ‘posterior’ part of the cluster (*Hoxd11*, *Hoxd12* and *Hoxd13*) along with the adjacent 5′ region, from the rest (*Hoxd10* to *Hoxd1*), thus allowing to evaluate the importance of regulatory influences associated with either the telomeric (Figure 1A, yellow) or the centromeric (Figure 1A, purple) neighborhoods of the cluster.

We first looked at the early expression of *Hoxd11* and *Hoxd10*, those genes immediately flanking the breakpoint. At E9, *Hoxd11* is normally transcribed in the most posterior aspect of the embryo, around the remnants of the primitive streak, as well as in adjacent mesoderm [22]. *In situ* hybridizations on mutant embryos carrying only an inverted cluster showed no detectable *Hoxd11* transcripts at this stage (Figure 1B). We examined progressively later developmental stages and, until 12.5 days, saw no transcription of *Hoxd11* in the trunk of mutant embryos (Figure 1B, lower panel). This effect was certainly more dramatic than the delay observed upon the loss of region VIII alone, a small DNA region that is deleted in one of the parental strains used for the inversion [14],[21]. *Hoxd11*, however, was expectedly expressed in the distal limb domain and the genital bud. These two domains were previously shown to depend upon late acting, global regulatory sequences lying centromeric to the cluster that kept the same relative position with *Hoxd11* in the inverted configuration [23],[24]. We then looked at both *Hoxd12* and *Hoxd13* and dramatic reductions in mRNA levels were scored (Figure 1C and D), suggesting that a long-range enhancer sequence, located on the telomeric side of the gene cluster, was required for the activation of these posterior *Hoxd* genes in the major body axis. Consequently, animals homozygous for the inversion lacked the functions of the three most posterior genes and expectedly displayed an anterior transformations of the sacral region (Figure S1A, B), thereby phenocopying the combined loss of function mutations of these three genes in *cis* [25],[26]. In contrast, and consistent with the observed gene expression in the developing distal limb, digits remained unchanged in this inversion.

### Repressive Effect from the Centromeric Side

Interestingly, however, this down-regulation of posterior *Hoxd* gene transcription could not be entirely explained by moving genes away from a potential activating sequence, for transgenic analyses of both the *Hoxd11* and *Hoxd12* loci had identified local *cis*-acting elements capable to elicit expression in the trunk when integrated randomly in the genome [27],[28]. These elements are present in the sub-cluster containing *Hoxd13*, *Hoxd12* and *Hoxd11* and their inability to function in the context of a split cluster thus suggested a negative effect exerted by the centromeric neighborhood over these transcription units. The analysis of *Hoxd10* and *Hoxd9* expression in the same mutant stock, at early stages, showed premature or elevated expression, respectively, consistent with these genes escaping such a repressive effect, due to their presence within the other sub-cluster, i.e. three Mb further apart (Figure 1E, F). Although this up-regulation was only transient, some mutant animals displayed clear skeletal abnormalities located at body levels much more anterior than the late expression boundaries of the corresponding genes (Figure S1C, D). The appearance of similar abnormal phenotypes after a transient gain of function was previously observed for the same gene, yet in a different genetic context [29]. Altogether, these data suggested the existence of a regulatory balance between a positive regulation, located telomeric to the cluster, and a repression, coming from the centromeric side, both acting on several genes and at a distance, to properly activate the *HoxD* cluster in the developing trunk.

### Transgene Scanning of the Activation Process

**Figure 2:**
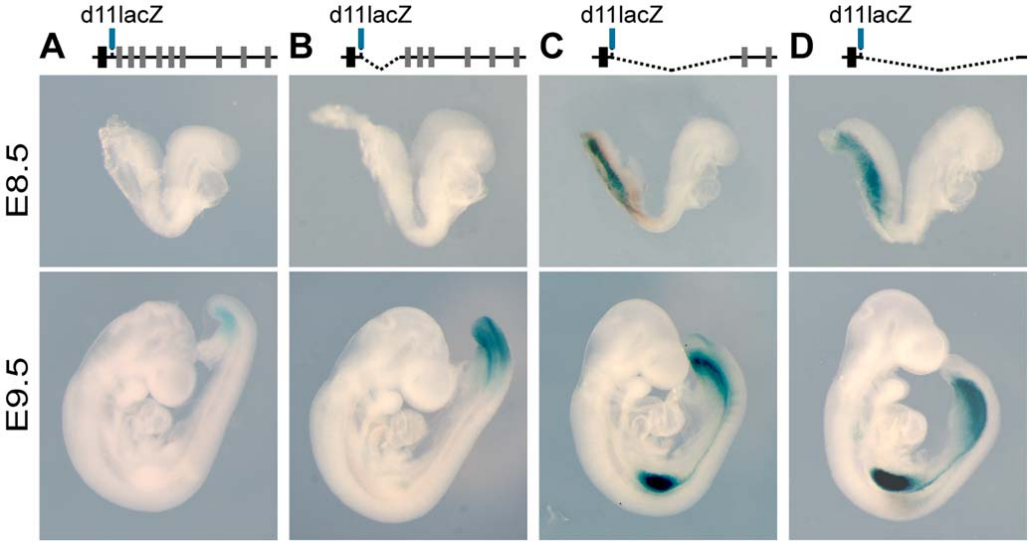
Transgene scanning of the *HoxD* cluster. (A–D) Expression patterns of the same *Hoxd11/lacZ* transgene after deletion of various DNA segments located telomeric from its insertion site. From left to right (schemes on the top): TgH[d11/lac], Del(11-13), Del(4-13) and Del(1-13). At E8.5 (top panels), only Del(4-13) and Del(1-13) (A–D) embryos show beta-gal expression in mesoderm derivatives. At E9.5 (lower panels), however, all configurations show staining in the primary body axis. Similar time dynamics are observed for transgene activation during limb outgrowth, such that only the Del(4-13) and Del(1-13) embryos are expressed in limb buds at E9.5 (C,D).

We challenged this view by looking at the timing of activation, *in vivo*, of a *Hoxd11*/lacZ reporter transgene positioned at various places along the gene cluster *via* successive *loxP*-dependent deletions (Figure 2). Following the above-mentioned hypothesis, the repressive effect *per se* exerted on the transgene should not be modified in such configurations, since only the relative distance to the activating sequence is progressively reduced. When placed within the *Evx2*-*Hoxd13* intergenic region, the transgene did not produce any signal at an early stage (Figure 2A, upper panel). Likewise, when a small deletion brought the transgene at the position of *Hoxd11*, no signal was scored (Figure 2B, upper panel). However, *lacZ* activity was detected whenever the transgene was placed further towards the telomeric extremity of the cluster, (Figure 2C and D, upper panels), well before the expected transcriptional onset for *Hoxd11* under normal conditions. Because the largest deletion had removed the entire cluster, leaving behind the *Hoxd11*/*LacZ* reporter transgene only, we concluded that at least part of the activation mechanism was located outside the complex (Figure 2D). Subsequently, however, all transgene relocations allowed for robust expression (Figure 2, lower panels), showing that the lack of early transcription was not caused by an inability to activate the transgene in a given context. Rather, it reflected a delay in the activation process.

### Deletion and Duplication Analyses

**Figure 3:**
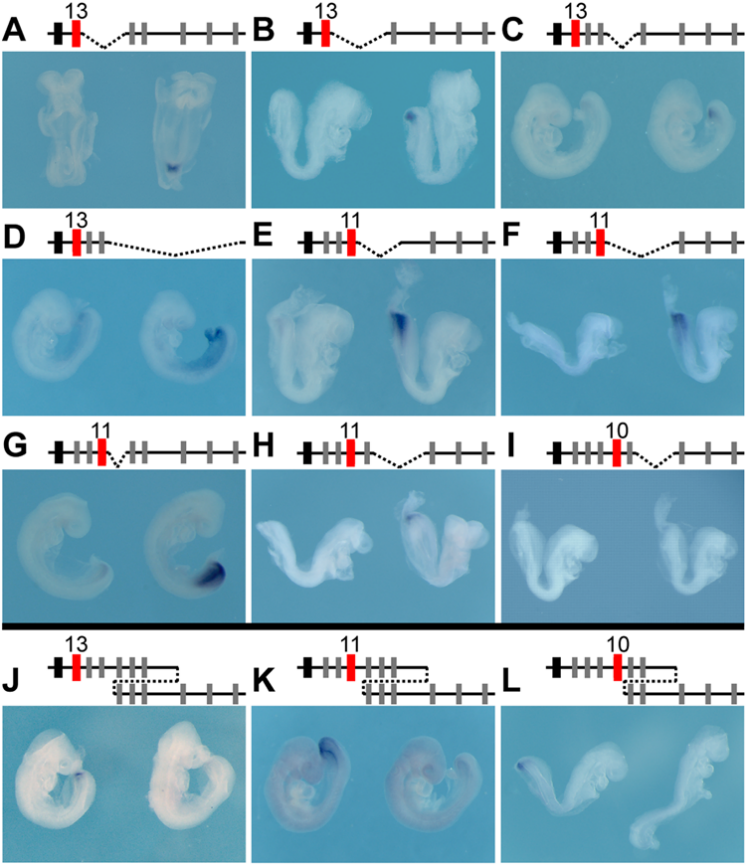
Expression onset in the trunk depends on the respective distance to the telomeric extremity. Mutant configurations are depicted on the top, with *Evx2* in black and the analyzed gene in red. Wild type embryos are always shown on the left of each panel, next to a representative mutant embryo, on the right. (A–I) E8 to E9 embryos hybridized either with a *Hoxd13* (A–D), a *Hoxd11* (E–H) or a *Hoxd10* (I) probe. In some cases, the gene tested is not directly neighboring the breakpoint, but lies further in 5′ (C, D, H and I). For all deletion alleles, expression is up-regulated in the mutant embryos, as compared to age-matched controls. The onset of expression was scored near the most posterior aspect of the embryo (e.g. *Hoxd13* in A). (K–M) Analysis of two duplication alleles, Dup(i-10) (J, K) and Dup(i-9) (L). E8.5 to E9 control (left) and mutant (right) embryos were hybridized with either *Hoxd13* (K), *Hoxd11* (L) or *Hoxd10* (M) probe and, in all three cases, signal accumulation was evident in control embryos, whereas undetectable in mutant specimens, indicating a delay in transcriptional activation for those genes that were relocated away from the telomeric end of the cluster.

We next looked at the impact of various deletions upon the activation timing of endogenous *Hoxd* genes located 5′ to the breakpoints, i.e. genes brought closer to the telomeric end of the cluster. In E8 to E9 embryos, a developmental window during which the most posterior *Hoxd* genes are normally silent, we systematically detected their premature transcription in the deleted configurations (Figure 3A–I). For example, any deletion which would bring *Hoxd13* closer to the 3′ end of the cluster led to its premature activation, regardless whether it was next to the breakpoint (Figure 3A, B) or further apart (Figure 3C, D). Similar effects were observed for *Hoxd11* (Figure 3E–H) and for *Hoxd10* (Figure 3I).

We then used two alleles carrying internal duplications and looked at the expression timing of those genes lying centromeric to the duplicated DNA segments. Three genes placed in such relative positions were analyzed and displayed a distinct delay in their transcriptional activation (Figure 3J–L). For example, the *cis*-duplication of the *Hoxd8* to *Hoxd10* DNA segment postponed activation of both *Hoxd11* (Figure 3K) and *Hoxd13* (Figure 3J). Here again, as for premature activations, several adjacent genes responded in a coherent manner to this regulatory re-allocation, suggesting the existence of a global, rather than local, mechanism of activation. Altogether, the relative position of a *Hox* gene within the *HoxD* cluster seems to largely determine its transcriptional timing in the primary body axis; the closer to the telomeric extremity, the earlier a gene was expressed in the developing trunk.

### Spatial versus Temporal Collinearities in the Trunk

**Figure 4:**
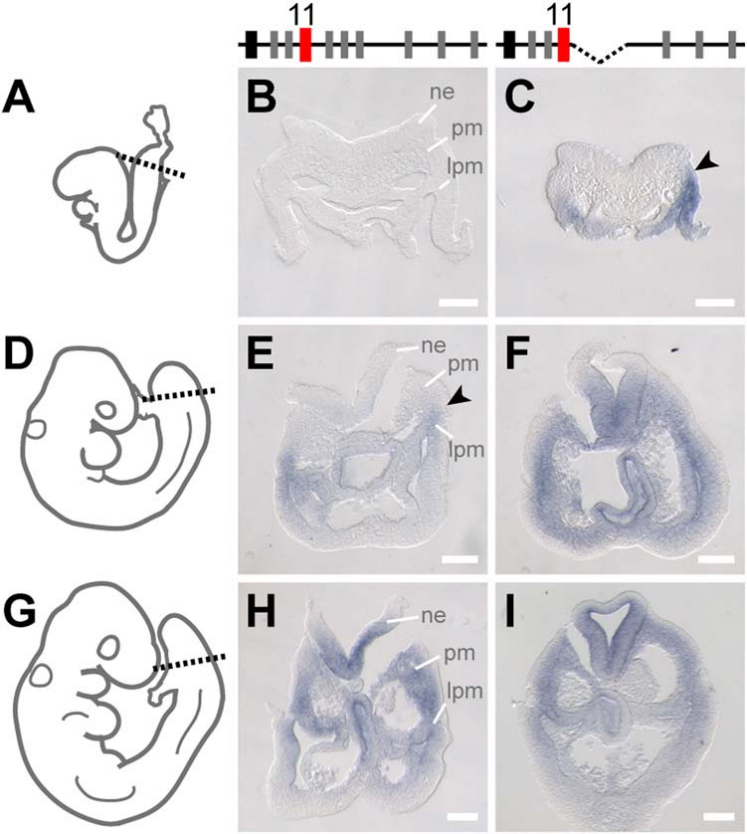
Premature activation of posterior *Hoxd* genes follows the wild type progression in tissue specificity. (A, D, G) Schemes of embryonic stages shown in the right panels. The orientations of section(s) are indicated by dashed line. Progression of the *Hoxd11* expression pattern, either in wild type (B, E, H), or in Del(8-10) mutant embryos (C, F, I). After initial activation at the posterior tip, expression is first scored as two lateral lines, within lateral plate mesoderm only, starting at about the dorso-ventral level of the junction between the future splanchnopleure and somatopleure (E, arrowhead). Subsequently, the gene becomes activated more dorsally, in paraxial mesoderm as well as in the neural tube (H). The same temporal sequence of tissue specificity is maintained for Del(8-10) mutant embryos, even though it was advanced in time (C, F, I). At E8.5 expression is indeed already apparent in mutant lateral plate mesoderm, whereas the wild type embryo is still devoid of any *Hoxd11* transcripts (compare C to B). Half a day later, expression has expanded into paraxial mesoderm and the neural tube in mutant embryos (F), while in wild type specimen transcripts just appear in lateral plate mesoderm (E). Both wild type and mutant embryos show the same *Hoxd11* expression pattern at E9.5, with lateral plate mesoderm, paraxial mesoderm and the neural tube all scoring positive (H, I). Bar is 50 mm. ne, neurectoderm; pm, paraxial mesoderm; lpm, lateral plate mesoderm.

To assess the relationships between the time of gene activation and the subsequent distribution of transcript in space, we re-visited the dynamics of *Hoxd* expression territories along the major body axis. The first transcripts were scored at the basis of the allantois, at the most posterior aspect of the gastrulating embryo (e.g. Figure 3A). Soon after, transcripts appeared in various mesoderm derivatives and in the neural plate, in a precise sequence that was best determined for *Hoxd10* to *Hoxd13*. In mesoderm, transcripts were first detected as two distinct lateral lines, matching the lateral plate mesoderm, rather than in PSM or in the neural plate. Positive cells were found from about the level of the joining of the splanchnopleural and somatopleural layers of the lateral plate mesoderm (Figure 4; arrowheads), slightly ventral to the intermediate (nephric) mesoderm whenever the section was rostral enough to identify this latter structure (not shown). Subsequently, however, expression of these posterior *Hoxd* genes was clearly observed within paraxial mesoderm, still in the presomitic areas, as well as in the adjacent spinal cord (Figure 4).

We investigated whether this generic progression in gene activation was conserved when the timing of activation was changed or, alternatively, if the mutant genomic context would modify tissue specificity along with the time variation. The general tendency is exemplified by the case of Del(8-10), where premature activation of *Hoxd11* was detected in the mesoderm of the body wall, yet not in the most dorsal cells (Figure 4). As for the wild type situation (but here in younger embryos), mesodermal expression was initially scored ventral to the pre-somitic mesoderm, whereas no transcripts were detected in neuro-epithelial cells. Subsequently, when *Hoxd11* appeared in the wild type embryo (Figure 4E), the mutant embryo, at a similar body level, was already positive for these transcripts in lateral plate mesoderm, in pre-somitic mesoderm as well as in the closing neural tube (Figure 4F). We concluded that premature *Hoxd* gene activation along the major body axis did not induce indiscriminate ectopic gene expression. Instead, premature activations followed the expected sequence in the detection of signals, within the various embryonic layers.

**Figure 5:**
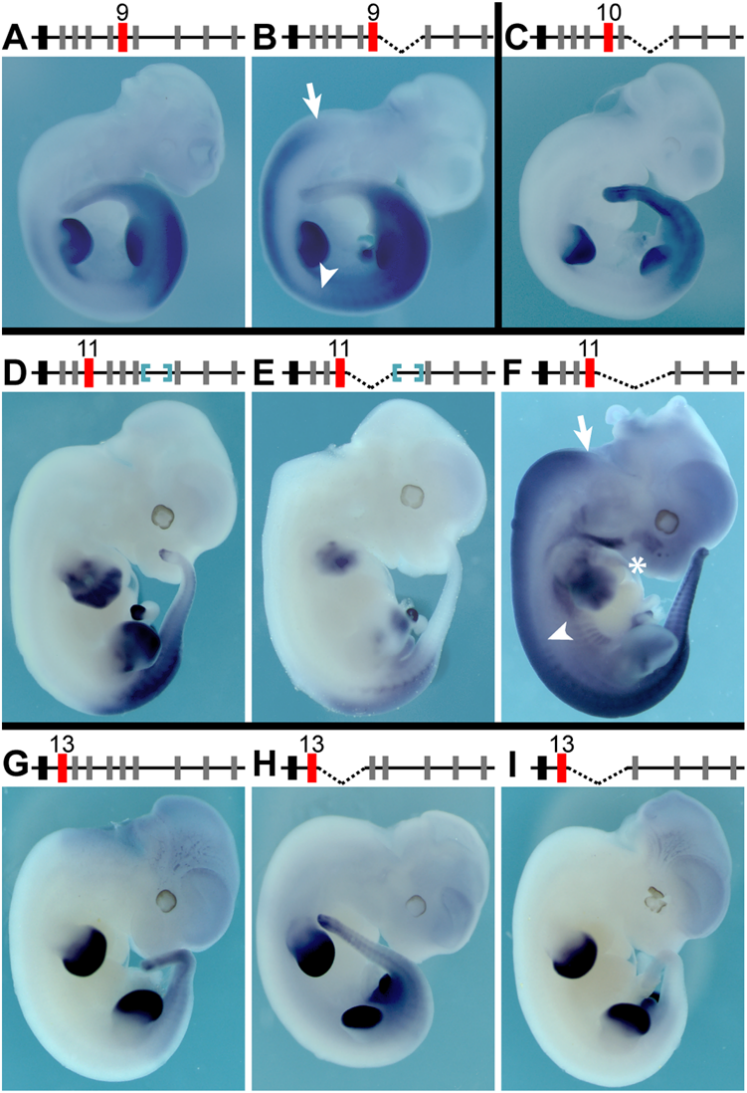
Spatial collinearity is independent of the timing of transcriptional onset. *Hoxd* gene expression patterns in a set of nested deletions. Schemes are as for Figure 3. (A–C) E10.5 wild type (A) and Del(i-8) mutant (B, C) embryos hybridized with a *Hoxd9* (A, B) or a *Hoxd10* (C) probe. *Hoxd9*, the gene neighboring the breakpoint shows both up-regulation and anteriorization in the neural tube (arrow) and the paraxial mesoderm (arrowhead) (B), whereas *Hoxd10*, located one transcription unit further away, follows its wild type expression patterns (C), unlike what was detected for the timing of activation. (D–F) E12.5 wild type (D) and mutant (E, F) embryos hybridized with a *Hoxd11* probe. Del(8-10) embryos have decreased *Hoxd11* expression levels in both the trunk and limbs, yet show largely wild type spatial distribution (E). Del(i-10) embryos display dramatic anterior gains of expression for *Hoxd11* in the neural tube (arrow), mesoderm derivatives (arrowhead) and branchial arch derivatives (asterisk) (F), caused by deleting the intergenic region “i” (in blue in schemes D, E). (G–I) E11.5 wild type (G) and mutant (H, I) embryos hybridized with a *Hoxd13* probe. While a down-regulation of *Hoxd13* is observed in the trunk of Del(9-12) embryos (I), no obvious difference with respect to wild type embryos is scored for Del(10-12) (H).

In marked contrast, no coherent impact on transcript distribution could be scored in our mutants, when analyzed at later stages. For example, the expression of both *Hoxd9* and *Hoxd11* was largely anteriorized, whenever the adjacent DNA was deleted up to the *Hoxd4* locus (Figure 5B, F). This was usually not the case for those genes located at more centromeric positions: while *Hoxd9* was clearly anteriorized in the Del(i-8) when placed near *Hoxd4* (Figure 5B), *Hoxd10* showed a wild type expression pattern in the same deletion (Figure 5C and data not shown), indicating that whatever the nature of the underlying mechanism is, it may act locally rather than at a global level. Two deletions sharing the same telomeric breakpoint confirmed this observation: firstly, *Hoxd11* was expressed too anteriorly in Del(i-10) mutant embryos, the ectopic domains recapitulating *Hoxd4* specific domains (Figure 5F).

Secondly, a shorter deletion leaving in place a gene-free DNA fragment (Del(8-10)) did not elicit the same response, even though the relative position of *Hoxd11* towards the telomeric part the cluster was as in the Del(i-10) allele (Figure 5E). In this case, the intergenic DNA fragment located between *Hoxd8* and *Hoxd4*, present in Del(8-10) but removed from Del(i-10), likely isolated *Hoxd11* from enhancers located around *Hoxd4*. Interestingly, these two deleted alleles displayed similar timing of premature activations (see Figure 3E and F). Therefore, while the effect of changing a gene's position upon its timing of activation was highly predictable, its subsequent spatial expression domain was impossible to anticipate. This observation was echoed by other alleles where neighboring gene expression was drastically reduced, if not abrogated. For example, the combined deletion of *Hoxd9* to *Hoxd12* led to the disappearance of *Hoxd13* expression in the tail and tailbud (Figure 5; compare G to I). However, when the extent of the deletion was slightly decreased, some expression was recovered (Figure 5H). More importantly, no anterior gain of expression was scored for either configuration, despite premature activation at earlier stages (compare to Figure 3A, B). Altogether, these spatial reallocations of transcript domains could be best explained by local, context-dependent modifications due to the effects of various breakpoints upon nearby-located enhancer sequences, rather than as direct consequences of the modified timing of activation.

### Centromeric Repression on the Deletion Alleles

**Figure 6:**
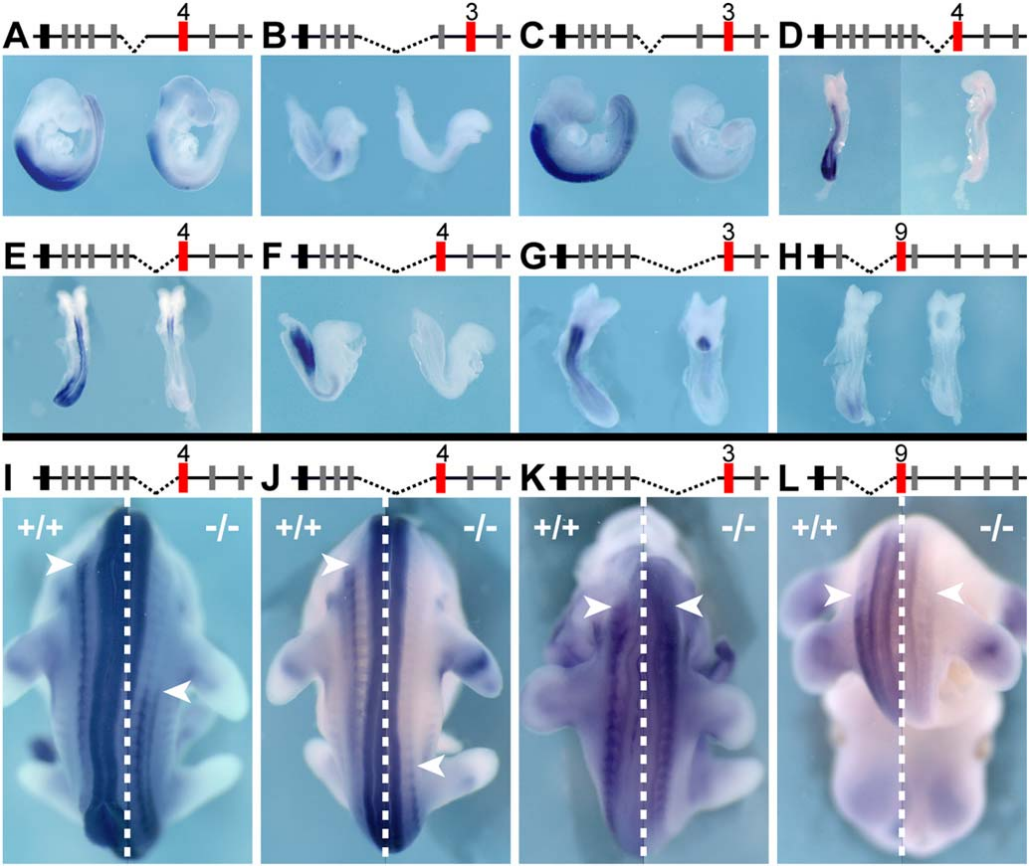
Attenuated transcription for *Hox* genes moved towards the centromeric end of the cluster. Expression patterns of *Hoxd3*, *Hoxd4* and *Hoxd9* in embryos carrying deletions in 5′ of these genes. Schemes and colors are as for Figure 3. (A–H) wild type (left) and mutant (right) embryos at E8–E9. Genes brought closer to the centromeric end of the cluster following a deletion systematically show reduced expression during early embryogenesis. A similar robust down regulation is observed in the mesoderm of Del(8-9) embryos for both *Hoxd4* (A) and *Hoxd3* (C), whereas expression persists in the anterior neural tube. In Del(i-10) mutant embryos (B, F) the signal for both *Hoxd4* (F) and *Hoxd3* (B) is decreased in the mesoderm as well as in the neural tube. Expression of the posterior gene *Hoxd9* disappears in pre-somitic mesoderm upon relocation closer to the centromeric side of the cluster (H). (I–L) Dorsal views of bisected and reconstituted pictures of wild type (left half) and mutant (right half) embryos at E11.5. Arrowheads demarcate anterior expression limits in the mesoderm. The same mutant alleles as in (E–H) are shown at a later developmental stage. Unlike for early embryos, no coordinated changes are observed. Expression of *Hoxd4* is posteriorized in the mesoderm of both Del(i-8) (I) and Del(i-10) (J). Changes in expression levels are observed for *Hoxd3* in Del(4-9) (K) and *Hoxd9* in Del(10-12) (L), yet both genes retain their wild type anterior expression boundary.

We also analyzed the expression dynamics of genes lying telomeric of various breakpoints in our deleted stocks. After deletions, these genes occupied relative positions closer to the ‘repressive influence’ emanating from the centromeric neighborhood, whereas their positions with regard to the telomeric side of the cluster remained unchanged. In E8 to E9.5 embryos, genes brought closer to the centromeric extremity *via* a deletion were consistently down-regulated, as exemplified by *Hoxd3*, *Hoxd4* and *Hoxd9* (Figure 6A–H). The same effect was scored for genes lying further away from the breakpoint, such as *Hoxd3* in the Del(i-10) and Del(8-9) (Figure 6B, C). Repression from the centromeric side contributed to this phenomenon, as transgenic approaches could exclude the deletion of distant promoters as the sole causative factor. Such transgenic analyses have defined local regulatory elements, as well as promoters, driving spatially correct expression for *Hoxd4* [30],[31]. Although these remained undisturbed, a clear down-regulation of *Hoxd4* was noticed (Figure 6A). Likewise, the observed weakening in *Hoxd9* transcription (Figure 6H, L) recalled an earlier observation whereby a *Hoxd9/LacZ* transgene was down-regulated when transposed into the *Evx2* to *Hoxd13* intergenic region [32].

Although we observed a clear posteriorization for *Hoxd4* in the mesoderm at later stages that became more pronounced the closer the gene was brought to the centromeric extremity (Figure 6I, J; arrowheads), other genes retained their anterior-posterior expression boundaries, yet changing their level of expression: in Del(9-4) mutant embryos, *Hoxd3* showed a slight but consistent increase of expression (Figure 6K), whereas a deletion sharing the same 3′ breakpoint (Del(11-4)) induced a decrease for the same gene (data not shown). Also, *Hoxd9* was down-regulated in the trunk when moved next to *Hoxd13* (Figure 6L). Expression analysis of genes located in 3′ of the breakpoints at these later stages thus did not reveal any coherent tendency. Rather, the diversity of the observed modifications pointed to independent, local regulatory reallocations, similar to what happened to *Hoxd* genes lying in 5′ of the respective breakpoints. Therefore, gene position with respect to either the centromeric, or telomeric extremities of the *Hoxd* gene cluster did not substantially affect spatial collinearity, in contrast to our observations regarding temporal collinearity.

## Discussion

### Does Time Fix Space?

Ever since collinearity was reported in vertebrates, pointing to a functional conservation between the way arthropods and vertebrates organize their body plans [4],[6], both the underlying molecular mechanisms and the nature of the associated evolutionary constraints have been discussed (see [16],[17],[33]). Differences in developmental strategies between diptera and vertebrates made it unlikely that the same genetic cascade would act upstream the *Hox* gene family. In search for an alternative mechanism, the observation of temporal collinearity, in vertebrates, suggested the timing of *Hox* gene activation as an important parameter in establishing the positions of the future transcript domains. However, while vertebrate *Hox* genes need to be clustered to properly achieve temporal control, clustering is not essential in all cases where spatial collinearity is observed (e.g. [15]). Here, we further challenged the causal link between temporal and spatial collinearities during trunk elongation in the mouse and we conclude that the final collinear distribution of *Hoxd* gene expression domains along the developing body axis is not strictly the function of their timing of activation during early development.

**Figure 7:**
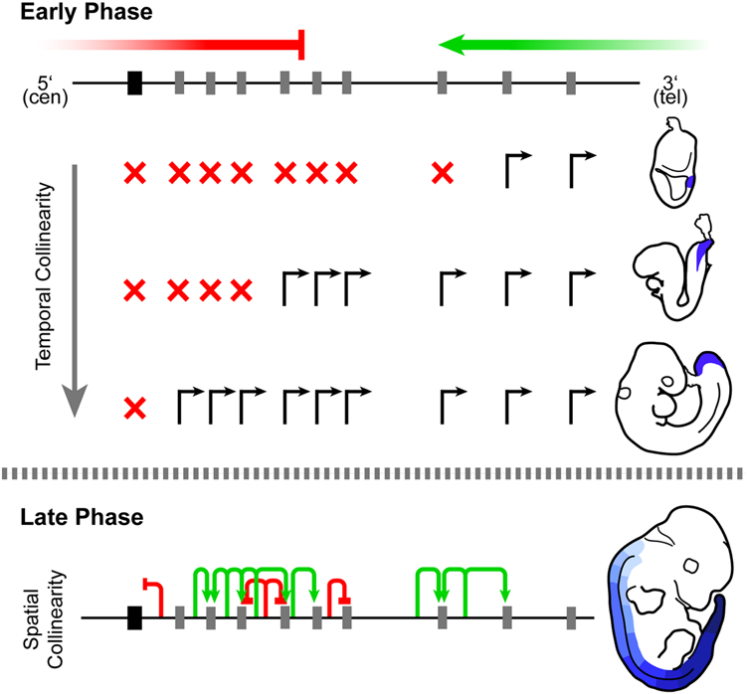
A two-phases model for the establishment of temporal and spatial collinearities of *Hoxd* genes in the trunk. Distinct mechanisms underlie the two collinear processes in the trunk. (A) In an early phase, a time-sequenced activation occurs, resulting from a balance between a repressive influence coming from the telomeric neighborhood (red), on the one hand, and a positive influence originating from the telomeric side (green), on the other hand. Initial activation starts from the telomeric side to subsequently expand over the entire length of the cluster in a 3′ to 5′ sequence. The gene's relative position within the cluster determines its timing of activation, in a distance-dependent manner. Active genes are shown with a black arrow, whereas silent genes are labeled with a red X. Approximate developmental stages are schematized on the right hand side with expression domains of the last activated genes highlighted in blue. (B) Subsequently (late phase), spatial transcript distribution along the anterior-posterior axis (as well as tissue-specificity) is determined by local regulatory elements, operating once the initial activation has occurred. Activating (green arrows) and repressive (red bars) elements can be shared by neighboring genes in a wild type situation. These elements can ectopically activate genes when relocated adjacent to them. The schematized E12.5 embryo on the right shows anterior (light blue) to posterior (dark blue) gene transcript distribution in both the neural tube and the paraxial mesoderm.

Our approach reveals a correspondence between the location of a gene relative to both extremities of the cluster and its timing of transcription, whereby proximity to the telomeric end is translated into precocity of activation. Accordingly, the onset of gene activation is likely controlled by a timing mechanism originating in the telomeric neighborhood of the *HoxD* cluster. Since this early mechanism seems to be shared by developing limb buds [11], we confirm the suggestion that it was co-opted from the trunk to tetrapod limb. However, unlike in developing limbs, we failed to see a coherent impact of our engineered heterochronies on the spatial distribution of transcripts along the anterior-posterior axis at later stages. At these stages, transcript distributions mostly depend upon local regulations, interspersed within the gene cluster, in marked contrast with the early events observed by using the same mutant strains, implying that different mechanisms exist for the early temporal and late spatial collinear processes in the trunk (Figure 7).

### Two Phases of *Hox* Gene Regulation in the Major Body Axis

These mechanistic differences support the existence of at least two distinct phases in the activation of *Hox* genes during axial development [8]; first a time-sequenced activation along the primitive streak and the node, controlled by globally acting opposite regulatory influences, followed by a second wave of activation controlled by local cues in tissues derived from these cells such as the various mesoderm derivatives and the neurectoderm. A biphasic activation [34],[35] could also explain why some early defects associated with temporal perturbations were transient and not carried along to later stages of development [32], as they only affect the early phase.

The mechanism involved in the late phase of activation may involve local effects such as enhancer sharing and/or competition [36], which could be easily disturbed in our genetic configurations leading to unpredictable outcomes. Regarding the early temporal activation, while global regulatory influences may rely upon remote enhancer sequences (e.g. [24]), they could as well involve, or be combined with-, processes such as chromatin modifications or chromosome looping [37]. For instance, the premature activations described in our set of deletions might reflect the successive removal of sequences, which evolved within the cluster to secure proper repression. While we do not rule out such a possibility, we think it can hardly account for some previously published results. In particular, a full inversion of the *HoxD* cluster lead to the premature activation of the inverted ‘posterior’ genes, even though, internally, the gene cluster remained untouched [38].

### Functions of Collinear Regulations

The respective functional contribution of each phase of activation to the primary body axis is unclear. In the mouse embryo, while the necessity to establish correct expression boundaries has been largely documented through various genetic approaches, the function of the early temporal sequence of activation is less explicit. Because this temporal process has been thus far associated only with animals where (an) integral *Hox* gene cluster(s) is (are) present, it may be one of the major constraints that kept *Hox* genes together. The analyses of additional animal species will be informative in this respect.

Both instructive and restrictive contexts can be considered (non-exclusively) when looking for the ‘raison d'être’ of temporal collinearity. In the former, a need exists for a precise time-sequence in the transcriptional activation of these genes and important direct functional outputs of this process may occur, perhaps at a time and in a cellular population that have so far escaped our analyses. An example of such an early mechanism is the observed delay in ingression, during gastrulation, of epiblast cells containing abnormal combinations of HOX proteins [39]. Alternatively, temporal collinearity simply illustrates the necessity, for the developing embryo, not to activate the most posterior *Hox* gene(s) too early, a situation detrimental to embryonic development. This is suggested both by the early lethality associated with the inversion of the complete *HoxD* cluster, *Hoxd13* becoming activated at the expected time for *Hoxd1* [38], and by the premature expression of *Hoxd10* and *Hoxd9* in the split cluster (this work). Whichever mechanism evolved to prevent the most posterior gene(s) to be expressed too early may have incidentally generated a graded timescale for those genes located in between and hence this series of genes is transcribed following their genomic order, without any particular functional relevance in itself.

### Fossil Regulations

The question as to which type of collinearity evolved first, i.e. whether the time-sequence preceded the spatial organization of the expression domains, or *vice versa* [40],[41] is concerned with the segmental status of the ancestral animal where this genetic system was implemented. If this animal indeed had a meristic organization, as a result of a time-sequenced addition of segments, it makes sense that temporal collinearity was already at work there and was then used as a ground for evolving spatial collinearity. In this case, particular collinear *Hox* expression domains found in animals having lost this developmental time sequence, such as in diptera, may have been progressively taken over by different regulatory mechanisms, disconnecting space from time (such as *gap* genes).

The evidence is compelling, however, that even animals containing an atomized *Hox* gene cluster still show reminiscences of spatial collinearity, suggesting that the timing mechanism was built on the top of an already constrained gene cluster. Altogether, we consider it unlikely that an animal species will ever be found, which contains a broken *Hox* gene cluster, develops following a simultaneous segmentation process and implements temporal collinearity. Accordingly, any species displaying a clear time sequence in the ontogeny of its metameric aspect should have an intact *Hox* cluster, associated with a transcriptional time-sequence. Also, it should not be taken for granted that the ancestral *Hox* gene collinear function will still be found in extant animal species. Different collinear mechanisms can co-exist with one another and the implementation of a collinear regulation may have paved the way for its replacement by a more efficient strategy. For example, a mere distance effect to a remote enhancer could set up a time sequence in the appearance of transcripts encoded by contiguous genes, a situation selected due to a particular adaptive value. Once in place, this genomic topology may facilitate the evolution of yet a different progressive regulation, for example the spreading of chromatin modifications. Over time, the accumulation of such secondary mechanisms could take over the initial constraint for these genes to remain clustered, making it possible for an ancestral mechanism to turn into a fossil regulation and disappear from this particular phylogenetic branch.

## Materials and Methods

### Mouse Strains and Crosses

The mutant strains used in this study, except for the Del(4-9) allele, were described previously: The inversion allele Inv(*Itga6*-*HoxD*rVIII) was obtained by sequential targeted recombination (STRING; [21]). The targeted *Hoxd11*-*lacZ* transgene TgH[d11/lac] and the associated Del(11-13), Del(4-13) and Del(1-13) were produced using *loxP*/Cre mediated site-specific recombination in ES cells [25],[32],[42],[43]. The remaining set of deletion and duplication alleles were all produced *in vivo* using targeted meiotic recombination (TAMERE; [44]: Del(1-10) [38]; Del(i-9), Del(8-10), Del(9-10), Del(10) [45]; Del(i-8), Del(i-10), Del(9-12), Del(10-12), Dup(i-9), Dup(i-10) [11]. The Del(4-9) allele was obtained by TAMERE, using as parental lines the Del(4-13) and L5, the latter strain carrying a single *loxP* sites between *Hoxd10* and *Hoxd9* [45]. Crosses were generally carried out using animals heterozygous for the respective alleles. For those crosses involving duplication alleles, the mother was heterozygous for a chromosome deficient for the gene to be analyzed such that +/Del embryos were used as control and Dup/Del as experimental embryos.

These experiments are in agreement with the Swiss law concerning animal protection. They are subject to an official authorization delivered by representative of the government.

### Genotyping

Genotyping was performed on isolated yolk sac DNA using either simplex or duplex PCR protocols. Mutant and control embryos were marked before performing WISH for subsequent identification. Embryos younger than E10 were re-typed after WISH, using standard DNA extraction procedures [46].

### *In Situ* Hybridization and Histology

Noon on the day of the vaginal plug was considered as E0.5. Embryos were dissected in PBS and fixed from 4 h to overnight in 4% PFA. Whole mount *in situ* hybridization (WISH) was performed according to standard protocols, with both mutant and control embryos processed in the same well to maintain identical conditions throughout the procedure. Probes were as before: *Hoxd3* [47], *Hoxd4* [48], *Hoxd8* [49], *Hoxd9* [50], *Hoxd10* and *Hoxd11* [51], *Hoxd12* [22], *Hoxd13* [52]. Whole mount detection of beta-galactosidase reporter activity was carried out as described [53]. Embryos were dissected in PBS and fixed shortly in 2% PFA for 5′ to 15′. For histology, embryos after WISH were cryoprotected in 30% sucrose and embedded in OCT compound. Sectioning was performed on a Leica CM1850 cryostat at 12–16 mm.

## Supporting Information

Figure S1 Phenotypic alterations in the axial skeleton of mice with a split *HoxD* cluster. Newborn animals were processed and stained for bone (alizarin red) and cartilage tissues (alcian blue). (A) Incidence of different lumbar vertebral formulae in wild-type, heterozygous and homozygous mutant animals. L5/6 and L6/7 indicate unilateral transformations of the first sacral vertebrae. (B) Complete transformation of the first sacral vertebra into a lumbar identity (S1>L7) in a homozygous mutant (right), as compared to the L6 formula observed in wild-type specimen (left) (C) Misalignment of the first rib to the sternum (#) and fusions of sternebrae four and five (*) in a homozygous mutant. (D) Seventh cervical vertebrae (C7) of heterozygous and homozygous animals showing ectopic bony material protruding from the transverse processes (arrowheads).
(1.45 MB TIF)

## References

[1] Duboule D, Morata G (1994). Colinearity and functional hierarchy among genes of the homeotic complexes.. Trends Genet.

[2] Krumlauf R (1994). Hox genes in vertebrate development.. Cell.

[3] Wellik DM, Capecchi MR (2003). Hox10 and Hox11 genes are required to globally pattern the mammalian skeleton.. Science.

[4] Duboule D, Dolle P (1989). The structural and functional organization of the murine HOX gene family resembles that of Drosophila homeotic genes.. Embo J.

[5] Gaunt SJ, Sharpe PT, Duboule D (1988). Spatially restricted domains of homeo-gene transcripts in mouse embryos: relation to a segmented body plan.. Development.

[6] Graham A, Papalopulu N, Krumlauf R (1989). The murine and Drosophila homeobox gene complexes have common features of organization and expression.. Cell.

[7] Lewis EB (1978). A gene complex controlling segmentation in Drosophila.. Nature.

[8] Deschamps J, van Nes J (2005). Developmental regulation of the Hox genes during axial morphogenesis in the mouse.. Development.

[9] Dolle P, Izpisua-Belmonte JC, Falkenstein H, Renucci A, Duboule D (1989). Coordinate expression of the murine Hox-5 complex homoeobox-containing genes during limb pattern formation.. Nature.

[10] Duboule D (1994). Temporal colinearity and the phylotypic progression: a basis for the stability of a vertebrate Bauplan and the evolution of morphologies through heterochrony.. Dev Suppl.

[11] Tarchini B, Duboule D (2006). Control of Hoxd genes' collinearity during early limb development.. Dev Cell.

[12] Whiting J, Marshall H, Cook M, Krumlauf R, Rigby PW (1991). Multiple spatially specific enhancers are required to reconstruct the pattern of Hox-2.6 gene expression.. Genes Dev.

[13] Puschel AW, Balling R, Gruss P (1991). Separate elements cause lineage restriction and specify boundaries of Hox-1.1 expression.. Development.

[14] Zakany J, Gerard M, Favier B, Duboule D (1997). Deletion of a HoxD enhancer induces transcriptional heterochrony leading to transposition of the sacrum.. Embo J.

[15] Seo HC, Edvardsen RB, Maeland AD, Bjordal M, Jensen MF (2004). Hox cluster disintegration with persistent anteroposterior order of expression in Oikopleura dioica.. Nature.

[16] Garcia-Fernandez J (2005). The genesis and evolution of homeobox gene clusters.. Nat Rev Genet.

[17] Monteiro AS, Ferrier DE (2006). Hox genes are not always Colinear.. Int J Biol Sci.

[18] Kmita M, Fraudeau N, Herault Y, Duboule D (2002). Serial deletions and duplications suggest a mechanism for the collinearity of Hoxd genes in limbs.. Nature.

[19] Kmita M, Duboule D (2003). Organizing axes in time and space; 25 years of colinear tinkering.. Science.

[20] Deschamps J (2007). Ancestral and recently recruited global control of the Hox genes in development.. Curr Opin Genet Dev.

[21] Spitz F, Herkenne C, Morris MA, Duboule D (2005). Inversion-induced disruption of the Hoxd cluster leads to the partition of regulatory landscapes.. Nat Genet.

[22] Izpisua-Belmonte JC, Falkenstein H, Dolle P, Renucci A, Duboule D (1991). Murine genes related to the Drosophila AbdB homeotic genes are sequentially expressed during development of the posterior part of the body.. Embo J.

[23] Gonzalez F, Duboule D, Spitz F (2007). Transgenic analysis of Hoxd gene regulation during digit development.. Dev Biol.

[24] Spitz F, Gonzalez F, Duboule D (2003). A global control region defines a chromosomal regulatory landscape containing the HoxD cluster.. Cell.

[25] Zakany J, Duboule D (1996). Synpolydactyly in mice with a targeted deficiency in the HoxD complex.. Nature.

[26] Spitz F, Gonzalez F, Peichel C, Vogt TF, Duboule D (2001). Large scale transgenic and cluster deletion analysis of the HoxD complex separate an ancestral regulatory module from evolutionary innovations.. Genes Dev.

[27] Gerard M, Duboule D, Zakany J (1993). Structure and activity of regulatory elements involved in the activation of the Hoxd-11 gene during late gastrulation.. Embo J.

[28] Herault Y, Beckers J, Kondo T, Fraudeau N, Duboule D (1998). Genetic analysis of a Hoxd-12 regulatory element reveals global versus local modes of controls in the HoxD complex.. Development.

[29] Kondo T, Duboule D (1999). Breaking colinearity in the mouse HoxD complex.. Cell.

[30] Zhang F, Popperl H, Morrison A, Kovacs EN, Prideaux V (1997). Elements both 5′ and 3′ to the murine Hoxd4 gene establish anterior borders of expression in mesoderm and neurectoderm.. Mech Dev.

[31] Morrison A, Ariza-McNaughton L, Gould A, Featherstone M, Krumlauf R (1997). HOXD4 and regulation of the group 4 paralog genes.. Development.

[32] van der Hoeven F, Zakany J, Duboule D (1996). Gene transpositions in the HoxD complex reveal a hierarchy of regulatory controls.. Cell.

[33] Lemons D, McGinnis W (2006). Genomic evolution of Hox gene clusters.. Science.

[34] Deschamps J, Wijgerde M (1993). Two phases in the establishment of HOX expression domains.. Dev Biol.

[35] Forlani S, Lawson KA, Deschamps J (2003). Acquisition of Hox codes during gastrulation and axial elongation in the mouse embryo.. Development.

[36] Sharpe J, Nonchev S, Gould A, Whiting J, Krumlauf R (1998). Selectivity, sharing and competitive interactions in the regulation of Hoxb genes.. Embo J.

[37] Chambeyron S, Bickmore WA (2004). Chromatin decondensation and nuclear reorganization of the HoxB locus upon induction of transcription.. Genes Dev.

[38] Zakany J, Kmita M, Duboule D (2004). A dual role for Hox genes in limb anterior-posterior asymmetry.. Science.

[39] Iimura T, Pourquie O (2006). Collinear activation of Hoxb genes during gastrulation is linked to mesoderm cell ingression.. Nature.

[40] Ferrier DE, Holland PW (2002). Ciona intestinalis ParaHox genes: evolution of Hox/ParaHox cluster integrity, developmental mode, and temporal colinearity.. Mol Phylogenet Evol.

[41] Duboule D (2007). The rise and fall of Hox gene clusters.. Development.

[42] Zakany J, Kmita M, Alarcon P, de la Pompa JL, Duboule D (2001). Localized and transient transcription of Hox genes suggests a link between patterning and the segmentation clock.. Cell.

[43] Zakany J, Duboule D (1999). Hox genes and the making of sphincters.. Nature.

[44] Herault Y, Rassoulzadegan M, Cuzin F, Duboule D (1998). Engineering chromosomes in mice through targeted meiotic recombination (TAMERE).. Nat Genet.

[45] Tarchini B, Huynh TH, Cox GA, Duboule D (2005). HoxD cluster scanning deletions identify multiple defects leading to paralysis in the mouse mutant Ironside.. Genes Dev.

[46] Mathieu J, Griffin K, Herbomel P, Dickmeis T, Strahle U (2004). Nodal and Fgf pathways interact through a positive regulatory loop and synergize to maintain mesodermal cell populations.. Development.

[47] Condie BG, Capecchi MR (1993). Mice homozygous for a targeted disruption of Hoxd-3 (Hox-4.1) exhibit anterior transformations of the first and second cervical vertebrae, the atlas and the axis.. Development.

[48] Featherstone MS, Baron A, Gaunt SJ, Mattei MG, Duboule D (1988). Hox-5.1 defines a homeobox-containing gene locus on mouse chromosome 2.. Proc Natl Acad Sci U S A.

[49] Izpisua-Belmonte JC, Dolle P, Renucci A, Zappavigna V, Falkenstein H (1990). Primary structure and embryonic expression pattern of the mouse Hox-4.3 homeobox gene.. Development.

[50] Zappavigna V, Renucci A, Izpisua-Belmonte JC, Urier G, Peschle C (1991). HOX4 genes encode transcription factors with potential auto- and cross-regulatory capacities.. Embo J.

[51] Gerard M, Chen JY, Gronemeyer H, Chambon P, Duboule D (1996). In vivo targeted mutagenesis of a regulatory element required for positioning the Hoxd-11 and Hoxd-10 expression boundaries.. Genes Dev.

[52] Dolle P, Izpisua-Belmonte JC, Brown JM, Tickle C, Duboule D (1991). HOX-4 genes and the morphogenesis of mammalian genitalia.. Genes Dev.

[53] Zakany J, Tuggle CK, Patel MD, Nguyen-Huu MC (1988). Spatial regulation of homeobox gene fusions in the embryonic central nervous system of transgenic mice.. Neuron.

