# Supplementary figures and images for: Uncoupling Time and Space in the Collinear Regulation of Hox Genes
Source: PLoS Genet. 2009 Mar 6;5(3):e1000398. doi: 10.1371/journal.pgen.1000398 (PMC2642670; doi:10.1371/journal.pgen.1000398)

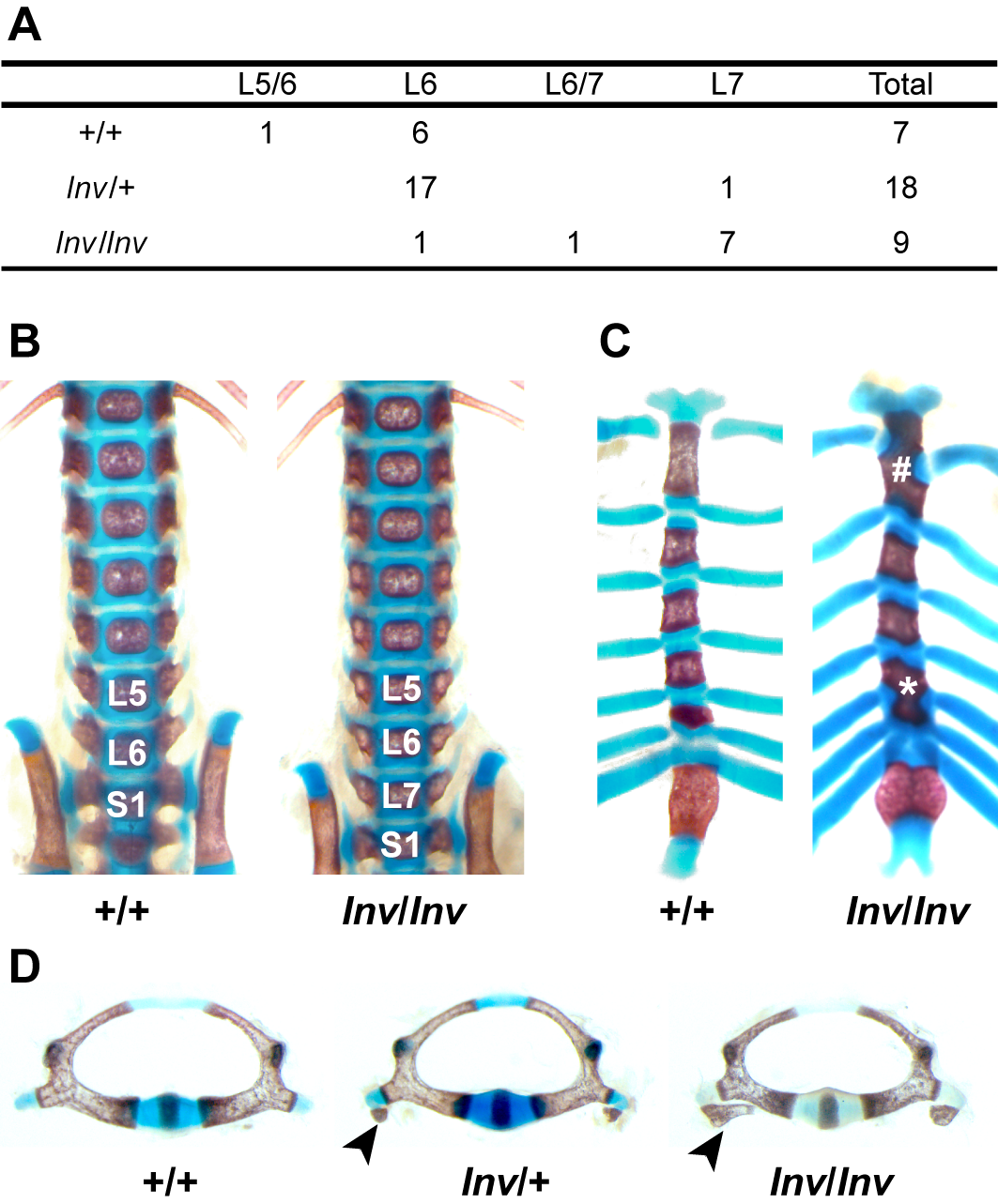

Supplement: Figure S1 — Phenotypic alterations in the axial skeleton of mice with a split HoxD cluster. Newborn animals were processed and stained for bone (alizarin red) and cartilage tissues (alcian blue). (A) Incidence of different lumbar vertebral formulae in wild-type, heterozygous and homozygous mutant animals. L5/6 and L6/7 indicate unilateral transformations of the first sacral vertebrae. (B) Complete transformation of the first sacral vertebra into a lumbar identity (S1>L7) in a homozygous mutant (right), as compared to the L6 formula observed in wild-type specimen (left) (C) Misalignment of the first rib to the sternum (#) and fusions of sternebrae four and five (*) in a homozygous mutant. (D) Seventh cervical vertebrae (C7) of heterozygous and homozygous animals showing ectopic bony material protruding from the transverse processes (arrowheads). (1.45 MB TIF) [file pgen.1000398.s001.tif]
